# Spontaneous intravesical sequestration of the adenomatous transition zone after prostatic artery embolization with Squid 12

**DOI:** 10.1186/s42155-026-00757-w

**Published:** 2026-08-12

**Authors:** Quentin Sénéchal, David Grinholtz, Marine Bravetti, Luisa Paulatto, Liess Laouisset

**Affiliations:** 1 Department of Interventional Radiology, Centre Cardiologique du Nord, Saint-Denis, 93200 France; 2 Department of Interventional Radiology, Hôpital Privé Paul d’Égine, Champigny-sur-Marne, 94500 France; 3 Department of Urology, CHP Sainte Marie, Osny, 95520 France

**Keywords:** Benign prostatic hyperplasia, Prostatic artery embolization, Squid, Ethylene–vinyl alcohol copolymer, Ischemic necrosis, Central gland detachment, Prostatic tissue sloughing, Intravesical sequestration

To the Editor

Prostatic artery embolization (PAE) is an established minimally invasive treatment for lower urinary tract symptoms (LUTS) attributed to benign prostatic hyperplasia [1]. Central gland detachment and spontaneous prostatic tissue elimination have been reported after particulate embolization, particularly in patients with marked intravesical median lobe protrusion [2, 3]. Recently, liquid embolic agents based on ethylene–vinyl alcohol copolymer (EVOH) have been explored for PAE because of their radiopacity, controlled delivery, and distal embolic penetration [4, 5]. We report intravesical sequestration of the adenomatous transition zone, containing polymerized EVOH, after PAE with Squid 12 (Balt, Montmorency, France).

A 68-year-old man, without diabetes, smoking, or anticoagulant use, was referred for severe LUTS refractory to alpha-blocker therapy and recurrent acute urinary retention. Prostate-specific antigen (PSA) was 22 ng/mL and multiparametric MRI showed no suspicious lesion (PI-RADS 2) but marked intravesical protrusion of an enlarged median lobe. Previous systematic biopsies had been negative. The prostate measured 56 cm^3^ and the International Prostate Symptom Score (IPSS) was 26/35.

Bilateral PAE was performed using Squid 12. A proximal EVOH plug was formed, and no angiographic evidence of non-target embolization was detected despite a distal pudendal anastomosis (Supplementary Fig. 1).

Acute urinary retention occurred on day 2, followed by an *Enterococcus faecalis* urinary tract infection. Over the following weeks, the patient developed persistent dysuria, recurrent urinary tract infections, exertional hematuria, and transient incontinence. These symptoms were initially managed as infectious and post-embolization complications, and no cross-sectional imaging was obtained during this interval. PSA decreased to 1.60 ng/mL at 6 weeks.

At 3 months, MRI showed regression of the median lobe with central cavitation resembling a resection cavity, and a large intravesical mass initially suspected to represent an intravesical blood clot (Fig. 1). CT urography demonstrated a hyperdense intravesical lesion composed of devitalized adenomatous tissue containing dense material consistent with polymerized EVOH (Fig. 2). Cystoscopy confirmed a freely mobile necrotic mass corresponding to en bloc detachment of the transition zone (Fig. 3; Video 1). Transurethral fragmentation and evacuation were performed. Histopathology confirmed extensive ischemic necrosis with embedded embolic material and no malignancy. Symptoms resolved. Six months after PAE, the patient was free of urinary retention, recurrent infection, and hematuria, with an IPSS score of 5/35.

**Fig. 1:**
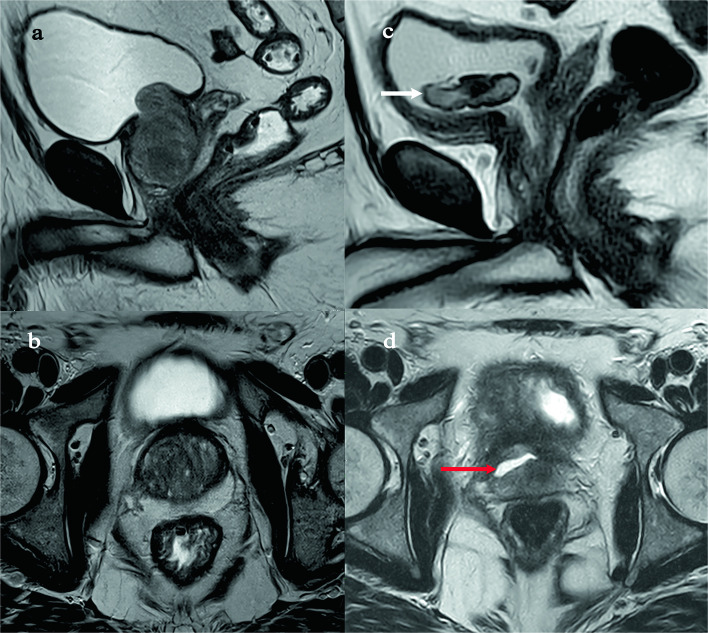
Prostate MRI before and after prostatic artery embolization. Preprocedural sagittal (**a**) and axial T2-weighted images (**b**) showing benign prostatic enlargement with nodules in the transition zone and intravesical protrusion of the median lobe. **c**, **d** Three-month follow-up MRI. Prostate volume had decreased to 15 cm^3^, corresponding to a 73% reduction. **c** A large intravesical mass is visible (white arrow), initially interpreted as clot retention. **d** Central cavitation (red arrow) with regression of the median lobe, resembling a transurethral resection cavity

**Fig. 2:**
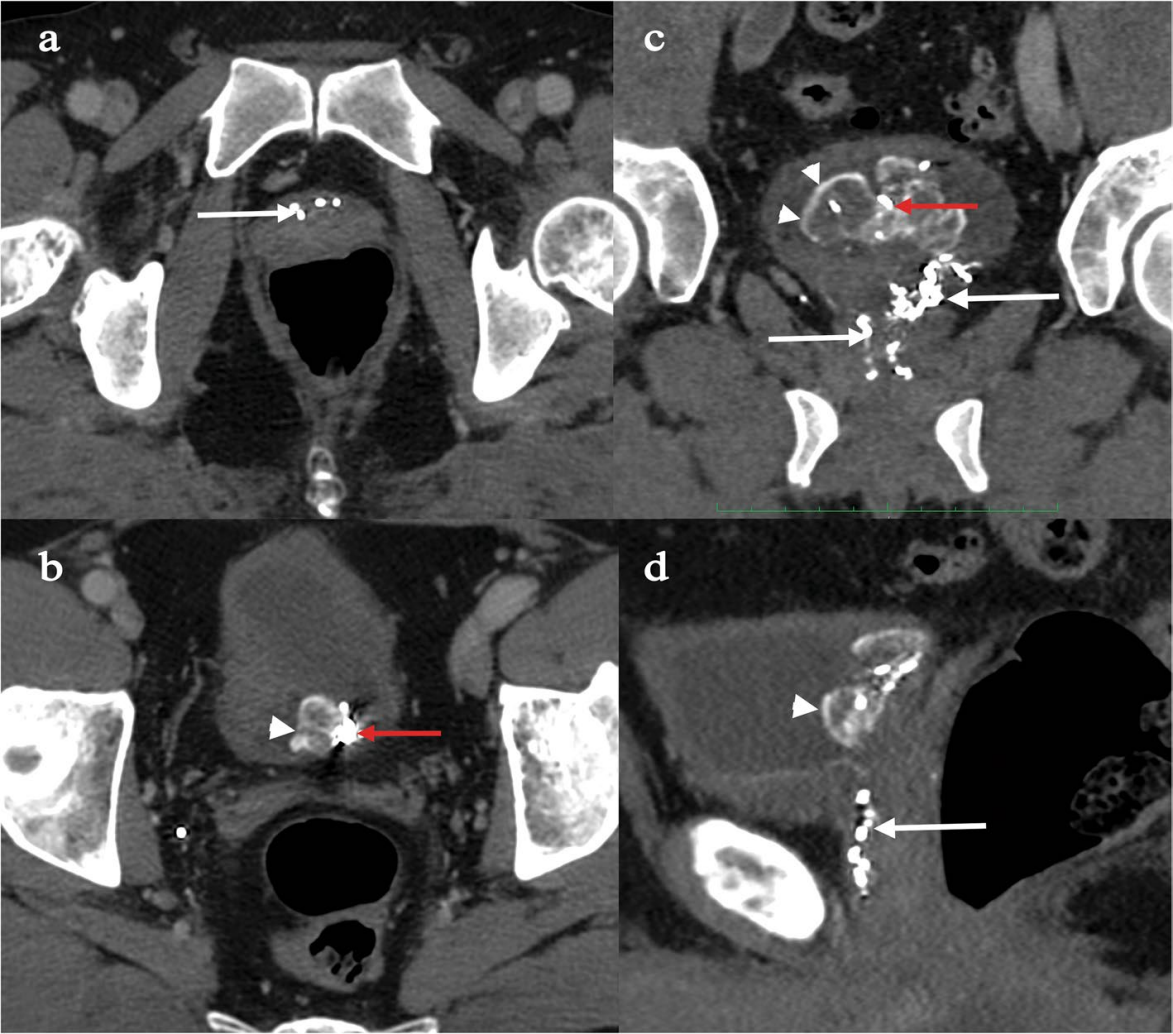
CT urography demonstrating intravesical sequestration of devitalized prostatic tissue. CT scan showing a hyperdense intravesical mass corresponding to a sequestrum arising from the adenomatous transition zone (arrowhead), containing dense polymerized embolic material (red arrow). Residual intraprostatic Squid cast is also visible (white arrow). **a**, **b** Axial images. **c** Coronal reconstruction. **d** Sagittal reconstruction

**Fig. 3:**
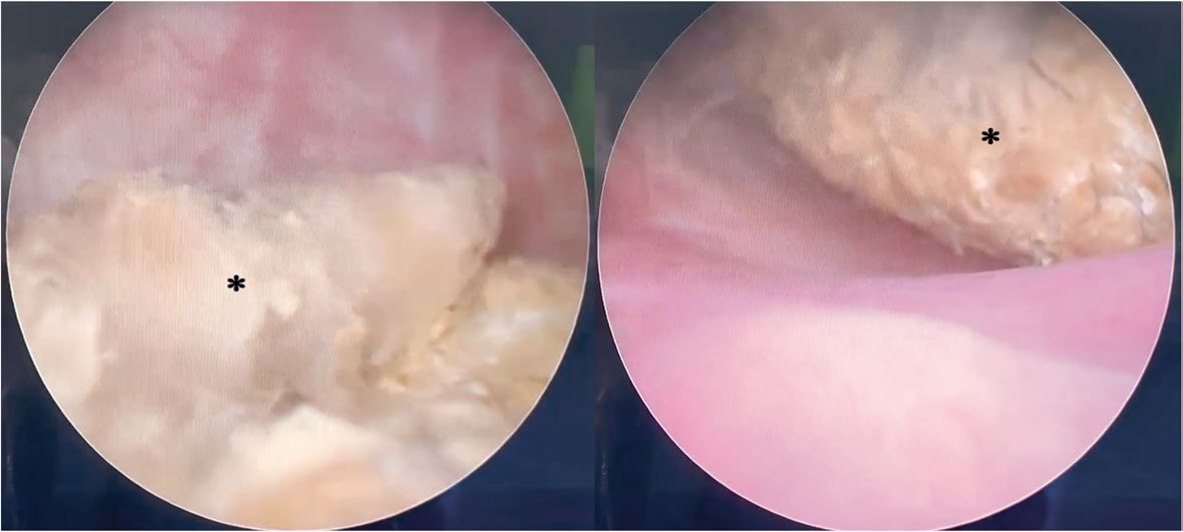
Cystoscopic view confirming a freely mobile necrotic intravesical mass before transurethral fragmentation and evacuation

Near-total volume reduction with transition-zone sloughing has been reported after PAE [6], including with particulate agents in patients with prominent median lobe protrusion. This anatomical risk factor was present in our patient. What has not, to our knowledge, been previously described is sequestration of an entire transition zone containing identifiable polymerized EVOH, raising the question of whether distal liquid embolic penetration may have contributed to the extent of ischemic necrosis or accelerated tissue detachment. This cannot be established from a single case, and a comparative assessment across embolic agents would be needed.

Persistent or worsening LUTS, recurrent infection, or hematuria after PAE, especially in patients with significant median lobe protrusion, should prompt early cross-sectional imaging rather than presumptive treatment for post-embolization syndrome, since delayed diagnosis of tissue sequestration may prolong avoidable morbidity.

## Supplementary Information

Additional file 1: Supplementary Video 1. Cystoscopic visualization and transurethral evacuation of the intravesical sequestrum. The video shows a freely mobile necrotic mass within the bladder lumen, corresponding to en bloc detachment of the adenomatous transition zone after prostatic artery embolization. Transurethral fragmentation and evacuation were performed.

Additional file 2: Supplementary Figure 1. Selective angiography during bilateral prostatic artery embolization using Squid 12. (a) Superselective left prostatic angiogram obtained through a microcatheter (black arrow), demonstrating intraprostatic arterial supply and a distal anastomosis with the internal pudendal circulation (white arrow; star indicates the pudendal artery). (b) Post-embolization image showing dense intraprostatic Squid cast (black arrow). (c) Superselective right prostatic angiogram demonstrating the prostatic arterial trunk supplying the central gland (white arrow).

## Data Availability

All data generated or analyzed during this study are included in this published article and its supplementary information files.

## Abbreviations

CT: Computed tomography
EVOH: Ethylene-vinyl alcohol copolymer
IPSS: International Prostate Symptom Score
LUTS: Lower urinary tract symptoms
MRI: Magnetic resonance imaging
PAE: Prostatic artery embolization
PSA: Prostate-specific antigen
